# Gastroprotective and anti-*Helicobacter pylori* potentials of essential oils from the oleoresins of *Araucaria bidwillii* and *Araucaria heterophylla*

**DOI:** 10.1007/s10787-022-01112-w

**Published:** 2022-12-21

**Authors:** Dalia E. Ali, Marwa M. Abd el-Aziz, Sherihan Salaheldin Abdelhamid Ibrahim, Eman Sheta, Essam Abdel-Sattar

**Affiliations:** 1 Department of Pharmacognosy and Natural Products, Faculty of Pharmacy, Pharos University in Alexandria, Alexandria, Egypt; 2 The Regional Center for Mycology and Biotechnology, Al-Azhar University, Cairo, Egypt; 3 Department of Pharmacology and Therapeutics, Faculty of Pharmacy, Pharos University in Alexandria, Alexandria, Egypt; 4 Department of Pathology, Faculty of Medicine, Alexandria University, Alexandria, Egypt; 5 Department of Pharmacognosy, Faculty of Pharmacy, Cairo University, El-Kasr El-Aini St, Cairo, 11562 Egypt

**Keywords:** Resin oil, *Araucaria*, Anti-*Helicobacter pylori* activity, Gastroprotective activity, Antioxidant effect, Anti-inflammatory effect

## Abstract

Plant resins or oleoresins comprise a chemically complex mixture of different classes of compounds. Oleoresin of the genus *Araucaria* combines essential oil (EO) and resin. It possesses gastroprotective, cytotoxic, and timicrobial, antipyretic, and anti-inflammatory activities. The study aimed to investigate the EOs from the oleoresins of two *Araucaria* species, *A. bidwillii* and *A. heterophylla,* chemically and biologically for their gastroprotective, anti-inflammatory, antioxidant, and anti-*Helicobacter pylori* potentials. The chemical composition of both species cultivated in Egypt was analyzed with GC-MS and compared with those cultivated abroad using principal component analysis (PCA). There were 37 and 17 secondary metabolites identified in *A. heterophylla* and *A. bidwillii*, respectively. The EOs of both species showed a pronounced inhibitory effect on Helicobacter pylori activity in vitro. The gastroprotective effect was assessed in vivo using ethanol-induced gastric ulcer model in rats. Inflammatory cytokines, oxidative stress, and the nuclear factor-kappa B (NF-κB) biomarkers were assessed in the stomach tissues. The ulcer index and percentage of ulcer protection were determined. Stomach sections were examined histopathologically by staining with (H/E) and periodic acid Schiff (PAS). Moreover, the proliferative index was determined using the Ki-67 immunostaining. The treatment of rats with EOs (50, 100, and 200 mg/kg, orally) 1 hour prior to ethanol administration showed promising gastroprotective, anti-inflammatory, and antioxidant potentials. These findings declared the gastroprotective role played by both EOs with the superiority of *A. bidwillii* over *A. heterophylla* via modulation of oxidative stress/NF-κB/inflammatory cytokines. Their use can be recommended to protect against the recurrence of peptic ulcers.

## Introduction

Gastric ulcer is one of the most common gastrointestinal problems that frequently occurs in old-aged individuals and men more than women (Aziz et al. 2019). It is characterized by damage of the stomach’s mucosa due to imbalance between aggressive (acid and pepsin) and defensive factors (mucosal defensive factors, prostaglandins (PG), and blood supply) (Paula de Oliveira et al. 2011). The imbalance mentioned above can be triggered by many factors, such as infection by *Helicobacter pylori*, ethanol abuse, psychological stress, smoking, and excessive or long-term use of nonsteroidal anti-inflammatory drugs (NSAIDs) (Chen et al. 2018).

The ethanol-induced gastric ulcer model is commonly used to investigate new agents’ gastroprotective effects. The pathophysiology of ethanol-induced gastric ulcer has been correlated with the interplay between several factors such as reactive oxygen species (ROS), nuclear factor-kappa B (NF-κB) activity, and inflammatory cytokines (Sidahmed et al. 2013; Takahashi et al. 2001). Ethanol intake may contribute to oxidative stress state which is accompanied by increase in the malonaldehyde (MDA) and decrease in the superoxide dismutase (SOD) levels. This may lead to the infiltration of neutrophils/macrophages and activation of the NF-κB transcription factor. Subsequently, the production of inflammatory cytokines such as tumor necrosis factor-alpha (TNF-α) and interleukin-1-beta (IL-1β) will be increased, leading to inflammation, ulceration, and damage of gastric mucosa (Arab et al. 2015).

Moreover, ethanol administration can reduce gastric blood flow, bicarbonate, and mucus secretion, which disturbs the balance between aggressive and defensive factors in gastric mucosa, leading to further mucosal damage (Raish et al. 2021). The Ki-67 is a nuclear protein expressed by proliferating cells but not by quiescent cells, so it is considered as a marker of proliferation and is an important issue for healing. It has been reported that Ki-67 expression was mitigated in the damaged mucosa (Fu et al. 2022).

Conventional pharmacological treatments for gastric ulcers, including proton pump inhibitors, histamine (H2) antagonists, and gastric mucosal protective agents, showed adverse drug effects, interactions, and relapse after discontinuation. Nowadays, the pursuit of discovering suitable gastroprotective herbal medicinal plants is of serious concern (Kuna et al. 2019).

Genus *Araucaria* is the most common genus with evergreen coniferous trees belonging to the family Araucariaceae. They are mainly used for ornamental timber (Aslam et al. 2013). The main constituents of the genus *Araucaria* are lignans, diterpenes, sesquiterpenes, phenylpropanoids, isoflavones, and bioflavonoids (Frezza et al. 2020). Members of the genus *Araucaria* species possess many pharmacological actions (Abdel-Sattar et al. 2009; Ali DE 2020; Schmeda-Hirschmanna et al. 2005; Verma et al. 2014). *A. heterophylla* (Salisb.) and *A. bidwillii* (Hook) are popular trees in the genus *Araucaria* and are known as Christmas trees (Norfolk Island pine) and dome-shaped trees (Bunya-bunya tree), respectively. It has been reported that the EOs from the oleoresins of both plants cultivated in Egypt possess anti-inflammatory and antipyretic effects in a carrageenan-induced rat model (Abdelhameed et al. 2021; Elshamy et al. 2020a, b). It was shown that the polyphenolic fraction from the leaves’ extracts of *A. bidwillii* could offer an anti-inflammatory effect in vitro using phytohaemagglutinin-stimulated human peripheral blood mononuclear cells (PBMCs) (Talaat et al. 2018). The methanolic leaves’ extracts of *A. heterophylla* and *A. bidwillii* possessed anti-inflammatory effects that could potentially play a role against COVID-19 (El-Hawary, Rabeh et al. 2021).

The current study aimed to investigate the unprecedented chemical composition of the EOs from the oleoresins of *A. bidwillii* and *A. heterophylla* cultivated in Egypt and evaluate the correlation between different *Araucaria* species resin worldwide. Moreover, the study assessed the EOs from the oleoresins of both species for their gastroprotective, anti-inflammatory, and antioxidant potentials in ethanol-induced gastric ulcer model using ranitidine as a standard drug. Also, the anti-*Helicobacter pylori* potential was assessed in vitro.

## Materials and methods

### Plant materials

Oleoresin of *A. heterophylla* was collected in April 2019 from El-Muntaza Palace Garden, Alexandria, while oleoresin of *A. bidwillii* was collected from the stem of the plants cultivated in the Giza Zoo, Giza, Egypt. The permission for the plant collection in April 2019 was obtained from the directors of both gardens. MS Therese Labib authenticated the plants, botanical specialist, and consultant at Orman Botanical Garden, Giza, Egypt. Voucher specimens of *A. heterophylla* (Sp. # AE 2.7.2019) and *A. bidwillii* (Sp. # AB 2.7.2019) were deposited at the Pharmacognosy Department, Faculty of Pharmacy, Cairo University, Egypt. The study complies with local, national, and international guidelines, and no specific consent was required to collect oleoresins from the plants.

### Essential oil extraction

The EOs of *A. heterophylla* and* A. bidwillii* oleoresins were prepared by the hydro-distillation method using a Clevenger-type apparatus for 5 h. The oily layer was separated and dehydrated over sodium sulfate anhydrous. Oils were stored in sealed air-tight glass vials at 4 °C until further analysis.

### GC-MS analysis

GC-MS spectra were recorded using Shimadzu GCMS-QP2010 (Koyoto, Japan) equipped with Rtx-5MS fused bonded column (30 m × 0.25 mm i.d. × 0.25 μm film thickness) (Restek, USA) equipped with a split–splitless injector. The initial column temperature was kept at 45 °C for 2 min (isothermal) and programmed to 300 °C at a rate of 5 °C/min and kept constant at 300 °C for 5 min (isothermal). The injector temperature was maintained at 250 °C. The flow rate of helium carrier gas was 1.41 ml/min. All the mass spectra were recorded applying the following condition: filament emission current, 60 mA; ionization voltage, 70 eV; ion source, 200 °C. Diluted samples (1% v/v) were injected with split mode (split ratio 1:15), and the injected volume was 1 µl.

### Identification of essential oil components

GC-MS data were processed using GC solution^®^ 2.4 (Shimadzu Corporation, Kyoto, Japan). Identification of the EOs components of both species was performed by comparing the mass spectra and their retention indices with spectra in NIST-11 database and by comparing with data published in the literature (Adams 2007; Elkady and Ayoub 2018; Elshamy et al. 2020a, b).

### Chemometric and principal components analysis (PCA)

Unscrambler^®^ X 10.4 software (from CAMO “Computer Aided Modeling”, Ås, Norway) was used to perform the principal component analysis (PCA) to evaluate the relation between EO components and to those reported from Indian species resin; *A. cunninghamii* and *A. heterophylla* (Verma et al. 2014) and Egyptian resin of *A. heterophylla (***(**Elshamy et al. 2020a, b**)**. In addition, we designed a matrix based on the concentration of 39 major compounds (≥ 1%).

#### In vivo study

### Animals

Seventy-two male Sprague–Dawley albino rats (150–175 g) were purchased from the animal house of the Faculty of Pharmacy, Pharos University in Alexandria. Rats were housed in cages with free access to food and water. All experimental procedures were approved by the “Unit of Research Ethics Approval Committee, Pharos University in Alexandria” (PUA-01202206263035). All procedures strictly followed the Guidelines for the Care and Use of Laboratory Animals (NIH) and the Animal Research: reporting of in vivo Experiments (ARRIVE) guidelines.

### Experimental animal design

**Fig. 1:**
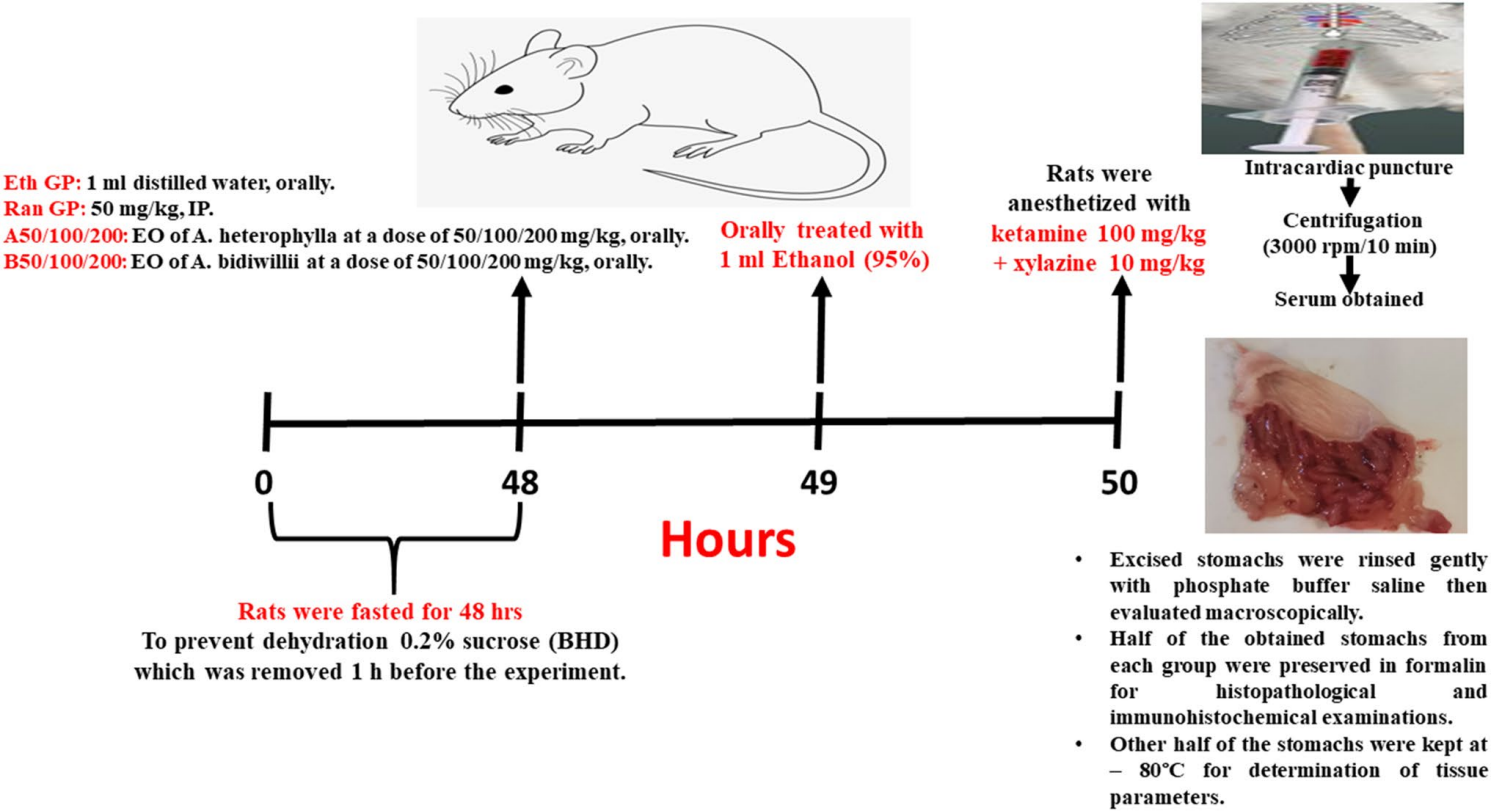
Experimental design for evaluating the gastroprotective effect of EOs of *A.* *heterophylla* and *A.* *bidwillii* in ethanol induced peptic ulcer model in rats

The experimental animal design is illustrated in detail in Fig. 1. The gastroprotective activity was evaluated in the ethanol-induced gastric ulcer model in rats according to the method described by (Ode and Asuzu 2011). After acclimatization, rats were fasted for 48 h before starting the experiment. To prevent dehydration during the fasting period, rats were supplied with 0.2% sucrose (BHD), which was removed 1 h before the experiment (Abdel-Sattar et al. 2009). The rats were randomly assorted into nine groups (eight rats/group). Group 1 (control): normal healthy animals served as the negative control group; Group 2 (ethanol control): the animals received distilled water in equal volume to that of EO given to other groups, and 1 h later received 95% ethanol (1 ml for each rat) (Elshamy et al. 2020a, b); Group 3 (ranitidine-treated group): animals received ranitidine (50 mg/kg, IP) as a standard drug (Elshamy et al. 2020a, b); Groups (4, 5, and 6): animals treated orally with *A. heterophylla* oil (AH-A) (50, 100, and 200 mg/kg, respectively); and Groups (7, 8, and 9): animals treated orally with *A. bidwillii* (AH-B) (50, 100, and 200 mg/kg, respectively). Ranitidine and EOs groups were treated 1 hour prior to oral intake of ethanol.

### Serum parameters

At the end of the experiment, the animals were anesthetized with ketamine 100 mg/kg and xylazine 10 mg/kg. Blood samples were collected via cardiac puncture, centrifuged (3000 rpm/10 min), where clear serum was separated and used for the determination of TNF-α (Cat no. MBS825075, My BioSource, USA) and IL1-β (Cat no. ab255730, Abcam, USA) by using ELISA kits according to the manufacturer’s instructions.

### Macroscopic assessment

Excised stomachs were rinsed gently with phosphate buffer saline (PBS) and then evaluated macroscopically to assess the ulcer area. The excised stomachs were photographed by a digital camera. Photos were analyzed using Image J software (NIH, Bethesda, MD) to calculate the ulcer index (Guzmán-Gómez et al. 2018). The ulcer index was determined by calculating the ratio of gastric ulcer area and total gastric area using the following equation:

$$\mathrm{The\,percentage\,of\,ulcer\,area }=\frac{\mathrm{Ulcer\,area}}{\mathrm{Stomach\,area}}\times 100\%$$

Then the percentage of ulcer protection was calculated according to (Elshamy et al. 2020a, b)

$$\mathrm{Ulcer\,protection }\left(\mathrm{\%}\right)=\frac{\mathrm{Ulcer\,index\,of\,control}-\mathrm{ Ulcer\,index\,of\,test}}{\mathrm{Ulcer\,index\,of\,control}}\times 100$$

### Oxidative stress parameters

The stomach tissues from all experimental groups were used to determine MDA (Cat no. MBS741034, My BioSource, USA) and SOD (Cat no. MBS034842, My BioSource, USA) by using colorimetric assay kits according to the manufacturer’s instructions.

### Western blot analysis

Samples of gastric tissues obtained from each group were homogenized and lysed with lysis buffer to perform a radioimmunoprecipitation assay (RIPA). Nuclear and cytoplasmic proteins were separated according to the protocol described in the Nuclear and Cytoplasmic Extraction Kit (Thermo Scientific, USA). Samples were separated by 10% sodium dodecyl sulfate–polyacrylamide gels (SDS-PAGE) and transferred to polyvinylidene fluoride membrane (PVDF). Furthermore, the membranes were blocked for 2 h with 5% skim milk in TBST buffer and probed with primary antibodies as follows: NF-κB p65 (1:2000, ab16502, Abcam, United Kingdom); IκBα Mouse mAb (dilution 1:1000; cat. no. 4814; Cell Signaling Technology, Inc.); phosphorylated (p)-IκBα rabbit mAb (dilution 1:1000; cat. no. 2859; Cell Signaling Technology, Inc.); and *β*-actin (1:1000, sc-47778, Santa Cruz, CA, United States) followed by secondary horseradish peroxidase-conjugated antibody. Immunolabeled proteins were detected by incubation with ECL substrate, and the gray density was measured. Target protein levels were normalized against the level of *β*-actin.

### Microscopic assessment of H & E and PAS stained sections

Representative stomach sections were then cut and processed into paraffin blocks. The H & E stained sections were assessed for any pathological changes. Semi-quantitative scoring was applied to examine sections, according to **(**Sibilia et al. 2003**)**. Mucosa lesions were assessed according to different parameters. First, the mucosa length was measured, where the percentage of damaged mucosa was measured as the percent of all examined tissue. Then the score was given as 0 = if no pathology was detected, 1: < 10%, 2: 11–20%, and 3 if > 20% of mucosa was damaged. The second parameter was the depth of mucosal damage. The whole mucosal thickness from the lumen to the level of the upper submucosa was measured using Image J software (NIH, Bethesda, MD), then the thickness of mucosal lesions was assessed. A score was given according to the involved depth: 0 = no lesion, 0.5 = superficial erosions, 1: at least 33% of mucosal thickness is affected, 2 = 33–66%, and 3 if > 67% is affected. A total score (out of 6) will be given to each rat by summation of both mucosal length and depth scores. PAS-stained sections were assessed to interpret the mucin secretion in different groups.

### Proliferative index by Ki-67 immunostaining

Expression was examined in gastric neck cells in regenerating mucosa close to the ulcerated mucosa (Brito et al. 2018). Multiple sections were cut and mounted on positively charged slides. They were stained by Ki-67 primary antibody (#30–9, rabbit monoclonal antibody, ready to use) using autostainer Ventana benchmark GX IHC system (ROCHE DIAGNOSTICS, SN 816255). Positive cells were detected by the presence of dark brown staining of the nucleus. The proliferative index was assessed by calculating the ratio of positive cells and the total amount of cells in ten high-power fields (HPFs).

#### In vitro study

### Evaluation of the anti-Helicobacter activity

The antibacterial activity of tested EOs against *Helicobacter pylori* was determined using a reference strain *of H. pylori* (RCMB 031124, ATCC 43504) obtained from the Regional Center for Mycology and Biotechnology (RCMB), Al-Azhar University, Cairo, Egypt.

The antibacterial activity against *H. pylori* was determined using a micro-well dilution method. The inoculum of *H. pylori* was prepared, and the suspensions were adjusted to 10^6^ CFU/ml. The EOs of AH-A, AB-B, and the standard drug (clarithromycin) were prepared in dimethyl sulfoxide (DMSO) in a dilution range of 125–0.24 µg/ml, and the assay was performed in a 96-well plate. Each well of the microplate included 40 μl of the growth medium (Brain Heart Infusion (BHI)) in 10% fetal bovine serum (FBS), 10 μl of inoculum, and 50 μl of the diluted compounds. Clarithromycin and DMSO are used as positive and negative controls, respectively. The plates were incubated at 37 °C for 3 days in 5% O_2_, 10% CO_2_, and 85% N_2_ atmosphere. Later, 40 μl of 3-(4, 5-dimethyl-thiazol-2-yl)-2,5-diphenyl-tetrazolium bromide (MTT) at a final concentration of 0.5 mg/ml, freshly prepared in water was added to each well and incubated for 30 min. The change to the purple color indicated that the bacteria were biologically active. The inhibition percentage was calculated using the given formula:

$$ \% {\text{Inhibition = }}\frac{{{\text{Abs Control{-} Abs}}\,{\text{Sample }}}}{{{\text{Abs}}\,{\text{Control}}}} \times 100 $$

The concentration of samples (inhibitors) required for 90% inhibition (MIC 90) was determined from corresponding dose–response curves. The MIC was taken to the lowest concentration, where no change of color of MTT was determined using an automatic ELISA microplate reader at 620 nm. All the tests were performed in triplicates, and the means (± standard deviation (SD)) were estimated (Bonacorsi et al. 2009).

### Statistical analysis

We used One-way ANOVA followed by a Tukey post hoc test for multiple comparisons to analyze the obtained data. Data were expressed as mean ± standard deviation (SD) of 8 observations. A *P* value less than 0.05 was considered significant.

## Results

### Chemical profiling and GC-MS analysis of Araucaria oils

**Fig. 2:**
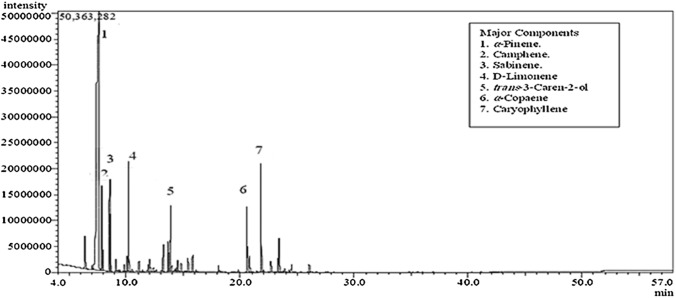
GC-MS chromatogram of essential oil from *A. heterophylla* resin collected from Egypt showing the major oil constituents; α-pinene (57.79%) followed by caryophyllene (5.4%), d-limonene (4.82%), and trans-3-caren-2-ol (4.65%)

**Fig. 3:**
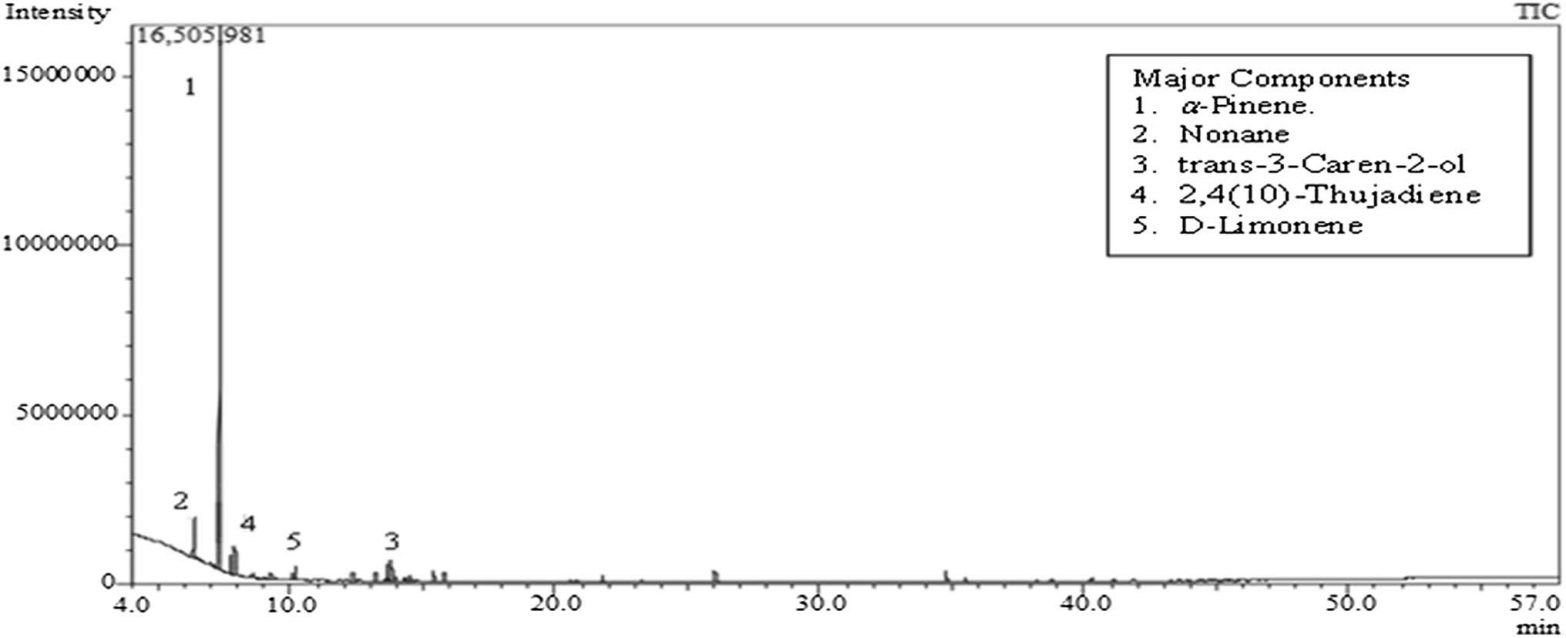
GC-MS chromatogram of essential oil from *A. bidwillii* resin collected from Egypt showing the major oil constituents; α-pinene (63.4%), nonane (5.21%), and trans-3-caren-2-ol (4.37%)

Hydro-distillation was performed using the Clevenger apparatus to extract oil from the oleoresin of the studied plants. The %yield was expressed as v/w% as follows: 5.6% v/w for the AH-A oil and 0.13% v/w for AB-B oil with a characteristic aroma. This amount was higher than that produced from *A. heterophylla* resin previously reported by Elshamy et al. **(**2020a, b) and approximately the same as that obtained from *A. heterophylla* oleoresin in India (Verma et al. 2014). Essential oil analysis using GC-MS of both species identified the presence of 37 components (97.53% of the total) in *A. heterophylla* (Fig. 2) and 17 components in *A. bidwillii* (90.78% of the total) (Fig. 3). Monoterpene hydrocarbons represented the major chemical class in both *A. heterophylla* (73.97%) and *A. bidwillii* (71.91%), followed by the oxygenated monoterpenes and sesquiterpene hydrocarbons. The qualitative and quantitative results of EOs from both oils are summarized in Table 1. AH-A oil was rich mainly in α-pinene (57.79%), followed by caryophyllene (5.4%), d-limonene (4.82%), and trans-3-caren-2-ol (4.65%). These results were close to the values previously reported for the Egyptian oil (α-pinene, germacrene-d, *α-*copaene, sabinene, and d-limonene with values of 44.88, 10.25, 4.72, 4.44, and 4.13%, respectively (Elshamy et al. 2020a, b). At the same time, *A. bidwillii* resin oil was enriched mainly with α-pinene (63.4%), nonane (5.21%), and trans-3-caren-2-ol (4.37%). On the contrary, beyerene, transnerolidol, and ɣ-elemene with concentrations of 35.65%, 13.66%, and 6.09%, respectively, were reported as the major components in the oil of Egyptian *A. bidwillii* leaves (AB-B) (Elkady and Ayoub, 2018). These findings confirmed that the oil composition is affected by the type of organ; therefore, a significant variation was noticed between the EO of the leaves and that of the resin from Egyptian species.

**Table 1:** Chemical profile of essential oils from *A. heterophylla* and *A. bidwillii* oleoresins

Identification*	Molecular formula	Content %		RI_lit_	RI_exp_	Compound	NO
		*A. Bidwillii*	*A. heterophylla*				
MS, RI	C_9_H_20_	5.21	2.25	900	899	Nonane	1
MS, RI	C_10_H_16_	–	0.26	905	903	Tricyclo[2.2.1.0(2,6)]heptane, 1,7,7-trimethyl-	2
MS, RI	C_10_H_16_	63.4	57.79	930	926	α-Pinene	3
MS, RI	C_10_H_16_	1.82	2.42	934	934	Camphene	4
MS, RI	C_10_H_14_	2.77	0.58	950	950	2,4(10)-Thujadiene	5
MS, RI	C_10_H_16_	–	3.6	970	970	sabinene	6
MS, RI	C_10_H_16_	–	1.75	973	973	β-Pinene	7
MS, RI	C_10_H_16_	–	0.45	990	990	β-Myrcene	8
MS, RI	C_10_H_22_	0.8	0.09	999	997	Decane	9
MS, RI	C_10_H_16_	–	0.23	1004	1005	( +)-4-Carene	10
MS, RI	C_10_H_14_	–	0.63	1014	1014	o-Cymene	11
MS, RI	C_10_H_14_	0.92	0.1	1028	1028	p-Cymene	12
MS, RI	C_10_H_16_	1.88	4.82	1029	1029	D-Limonene	13
MS, RI	C_10_H_16_	–	0.55	1056	1056	γ-Terpinene	14
MS, RI	C_10_H_16_	–	0.7	1088	1087	α Terpinolene	15
MS, RI	C_10_H_18_O	–	0.35	1098	1098	trans-Sabinene hydrate	16
MS, RI	C_11_H_24_	1.29	–	1100	1100	Undecane	17
MS, RI	C_10_H_14_	0.32	–	1113	1111	p-Mentha-1,5,8-triene	18
MS, RI	C_10_H_16_O	1.36	1.04	1125	1125	α-Campholenal	19
MS, RI	C_10_H_16_O	2.16	1.14	1129	1129	trans-Sabinol	20
MS, RI	C_10_H_16_O	–	0.25	1139	1139	α-Phellandren-8-ol	21
MS, RI	C_10_H_16_O	–	0.13	1164	1164	trans-Pinocamphone	22
MS, RI	C_10_H_18_O	–	0.08	1180	1180	α-Terpineol	23
MS, RI	C_10_H_14_O	–	0.74	1195	1195	Myrtenal	24
MS, RI	C_10_H_14_O	–	0.7	1205	1208	Verbenone	25
MS, RI	C_10_H_16_O	–	0.18	1220	1219	Carveol	26
MS, RI	C_10_H_16_O	4.37	4.65	1314	1314	trans-3-Caren-2-ol	27
MS, RI	C_15_H_24_	–	0.15	1361	1360	α-Ylangene	28
MS, RI	C_15_H_24_	0.55	2.75	1366	1366	α-Copaene	29
MS, RI	C_15_H_24_	0.56	0.69	1384	1384	(-)-β-Bourbonene	30
MS, RI	C_15_H_24_	1.05	5.4	1410	1410	Caryophyllene	31
MS, RI	C_15_H_24_	–	0.44	1444	1444	Humulene	32
MS, RI	C_15_H_24_	–	0.2	1459	1457	β-copaene	33
MS, RI	C_15_H_24_	–	0.58	1477	1477	ɣ-Muurolene	34
MS, RI	C_15_H_24_	–	0.03	1478	1478	Eudesma-4(14),11-diene	35
MS, RI	C_15_H_24_	–	0.08	1491	1490	ɣ-Amorphene	36
MS, RI	C_15_H_24_	–	0.18	1491	1490	α-Muurolene	37
MS, RI	C_15_H_24_	–	1.53	1503	1505	ɣ-Cadinene	38
MS, RI	C_15_H_24_	–	0.02	1532	1532	δ-Cadinene	39
MS, RI	C_20_H_30_O	0.61	–	2330	2330	Kaur-16-en-19-al	40
MS	C_20_H_30_O_5_	1.71	–	–	2630	Andrographolide	41
		71.91	73.97			Monoterpenes hydrocarbons	
		7.89	9.26			Oxygenated monoterpenes	
		2.16	12.05			Sesquiterpenes hydrocarbons	
		–	–			Oxygenated sesquiterpenes	
		2.32	–			Oxygenated diterpene hydrocarbons	
		6.5	2.25			Hydrocarbons	
		90.78	97.53			Total identified	

*The identification of EO constituents based on the comparison of the mass spectral data and Kovats indices (KI) with those of NIST Mass Spectral Library (2011) and Wiley Registry of Mass Spectral Data 8th edition and literature

^a^RI_lit_: published Kovats retention indices

^b^RI_exp_: Kovats index determined experimentally relative to C_8_–C_28_ n-alkanes

### Chemometric analysis

**Fig. 4:**
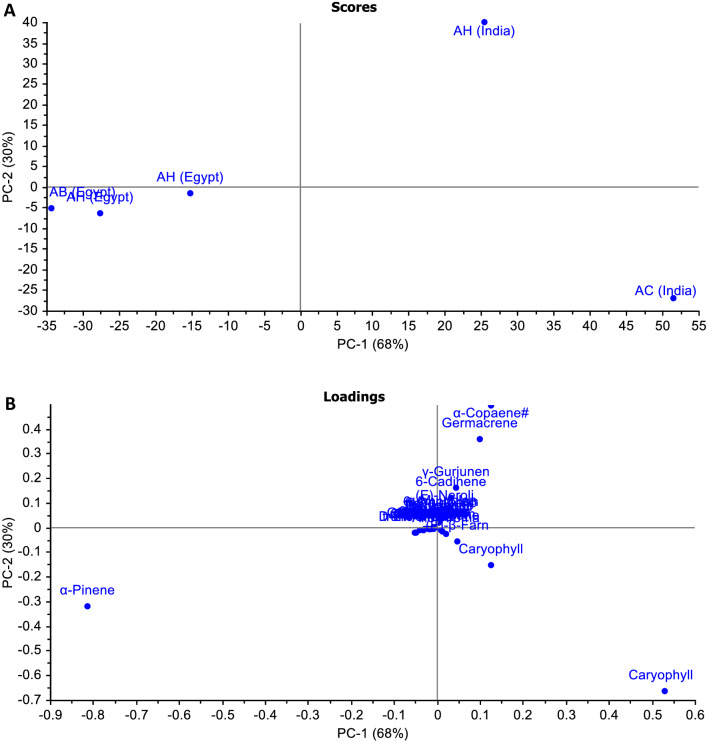
**A** PCA score plot and **B** loading plot of GC-MS analysis of essential oil of *A. heterophylla* & *A. bidwillii* from Egypt and *A. cunninghamii* & *A. heterophylla* from India showing the variation in the composition of the oils between the Indian and the Egyptian species

Chemometric analysis using PCA with GC-MS presents a successful method for finding the relationship between closely related compositions of EOs of the different* Araucaria* species resin worldwide. PCA was used to categorize samples, detect outliers, and find relationships between the tested samples and their variables. From the score plot, it was clear that the impact percentage of the 1st two principal components (68% for PC1 and 30% for PC2) clarifies 98% of the variance in the data (Fig. 4A). The score plot of PCA showed a clear separation between *A. cunninghamii* and *A. heterophylla* in Indian species, which were distributed towards the right of the plot (positive PC1 values). From the loading plot, the most influential EO components for this separation were caryophyllene oxide, germacrene, and *α*-copaene (Fig. 4B). However, the *Araucaria* species cultivated in Egypt are clustered close to each other at the other side (left side) of the vertical line representing PC1 (negative PC1 values), which accounted for 68% of the variance (Fig. 4A). This cluster’s key markers were α-pinene, camphene, d-limonene, and caryophyllene (Fig. 4B). The variation in the composition of the oils between the Indian and the Egyptian species could be attributed to many factors; biogenetic factors, geographical differences, besides the variation in the atmospheric conditions. In addition, agreement was shown between the current study’s findings and those reported previously by (Elshamy et al. 2020a, b).

#### Biological assessment

### Effect on serum inflammatory cytokines levels

**Fig. 5:**
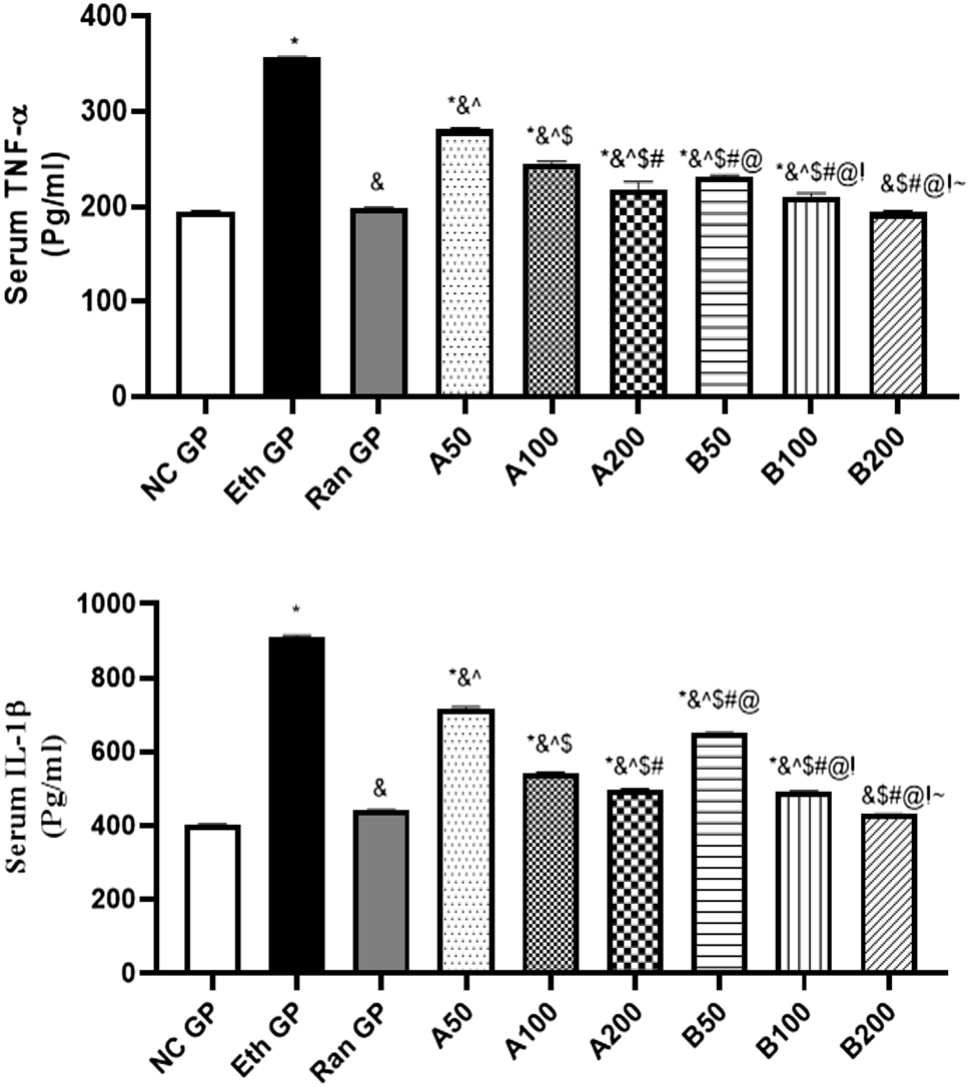
The reduction in the serum levels of TNF-α and IL-1β after pretreatment with Ranitidine or Essential oils of *A. heterophylla* (**A**) or *A. bidiwillii* (**B**) at 3 different doses (50, 100, 200 mg/Kg, oral) 1 h before ethanol (1 ml of 95%, oral) in ethanol induced peptic ulcer in rats. Each point represents the mean ± SD of 8 rats. Statistical significance: * is significantly different from NC GP, *P* < 0.05. & is significantly different from the Eth GP, *P* < 0.05. ^ is significantly different from Ran GP, *P* < 0.05. $ is significantly different from A50 treated group, *P* < 0.05. # is significantly different from A100 treated group, *P* < 0.05. @ is significantly different from A200 treated group, *P* < 0.05. ! is significantly different from B50 treated group, *P* < 0.05. ~ is significantly different from B100 treated group, *P* < 0.05, using one-way ANOVA followed by Tukey–Kramer multiple comparisons post-hoc test. *NC GP* normal control group, *Eth GP* ethanol treated group, *Ran GP* ranitidine treated group, *A50/100/200* EO of *A. heterophylla* at a dose of 50/100/200 mg/kg, *B50/100/200* EO of *A. bidiwillii* at a dose of 50/100/200 mg/kg

The changes observed in the serum TNF-α and IL-1β levels are illustrated in Fig. 5. Our study showed a significant elevation in the serum TNF-α levels in the peptic ulcer untreated group compared to the normal control group (356.3 ± 1.14 vs. 194.8 ± 0.86, respectively). The rats that were protected by the administration of ranitidine before ethanol administration showed low serum TNF-α levels reaching 197.8 ± 1.54, which was not significantly different from normal control rats.

Treatment with EO of *A. heterophylla* (AH-A) in variable doses (50, 100, and 200 mg/kg) significantly diminished the serum TNF-α levels reaching 280.9 ± 2.03, 245.8 ± 2.09, and 218.1 ± 8.13, respectively. At the same time, treatment with EO of *A. bidwillii* (B) with the same doses mentioned above mitigated the serum TNF-α levels reaching 231 ± 1.95, 210.3 ± 3.77, 193.7 ± 1.98, respectively. The AB-B oil at a 200 mg/kg dose offered the best anti-inflammatory effect, which was not significantly different from that of ranitidine treatment.

The serum IL-1β levels showed the same pattern of results as obtained for serum TNF-α. There was a significant elevation in the serum IL-1β levels in the peptic ulcer untreated rats by 2.27 fold compared to normal rats. Concomitant treatment with ranitidine before ethanol administration led to a significant reduction in the serum IL-1β levels by 51.44% compared to the ethanol group. Furthermore, treatment with AH-A oil prior to ethanol intake at doses of 50, 100, and 200 mg/kg mitigated the inflammatory parameter serum level by 21.04%, 40.49%, and 45.20%, respectively. Even though treatment with EO of AB-B before ethanol administration offered a much better decline in the serum IL-1β level at a dose of 200 mg/kg, reaching 52.57% as compared to the other two doses (50 and 100 mg/kg), reaching 28.42% and 47.99%, respectively.

### Macroscopic assessment of the collected stomachs from studied groups

**Fig. 6:**
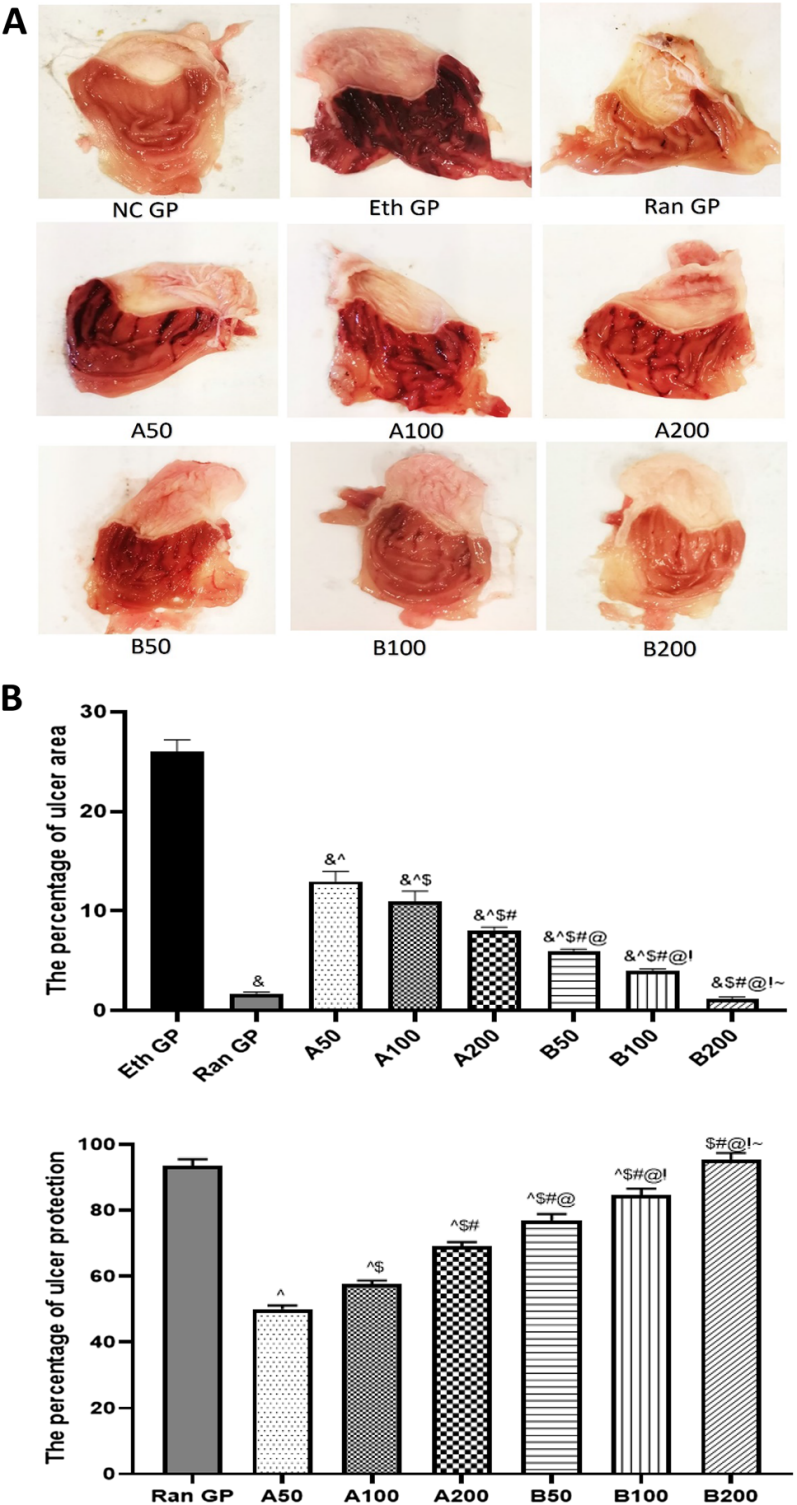
**A**, **B** The macroscopic assessments of excised stomachs as well as the change percentage of ulcer area and ulcer protection after pretreatment with ranitidine or Essential oils of *A*. *heterophylla* (**A**) or bidiwillii (**B**) at 3 different doses (50, 100, 200 mg/Kg, oral) 1 h before ethanol (1 ml of 95%, oral) in ethanol induced peptic ulcer in rats. Each point represents the mean ± SD of 8 rats. Statistical significance: * is significantly different from NC GP, *P* < 0.05. & is significantly different from the Eth GP, *P* < 0.05. ^ is significantly different from Ran GP, *P* < 0.05. $ is significantly different from A50 treated group, *P* < 0.05. # is significantly different from A100 treated group, *P* < 0.05. @ is significantly different from A200 treated group, *P* < 0.05. ! is significantly different from B50 treated group, *P* < 0.05. ~ is significantly different from B100 treated group, *P* < 0.05, using one-way ANOVA followed by Tukey–Kramer multiple comparisons post-hoc test. *NC GP* normal control group, *Eth GP* ethanol treated group, *Ran GP* ranitidine treated group, *A50/100/200* EO of *A. heterophylla at* a dose of 50/100/200 mg/kg, *B50/100/200* EO of *A. bidiwillii* at a dose of 50/100/200 mg/kg

Macroscopic assessment of excised stomachs from all groups is shown in Fig. 6A. The percentage of ulcer index/area and ulcer protection is illustrated in Fig. 6B. The normal control group showed intact mucosal folds with no congestion or edema. The mucosa was glistening and intact. Ethanol-treated group showed severe hyperemia of gastric rugae. Linear hemorrhagic ulcers were frequently seen, occupying 26% of the total area. The pretreatment by EO of AH-A showed improvement of ulcer index to be 13%, 11%, and 8% for 50, 100, and 200 mg/kg doses, respectively.

Meanwhile, the EO of AB-B pretreated groups showed an ulcer index of 12% and 7% in the 50 and 100 mg/kg doses, respectively. The best protective effect was seen in the group treated with oil of AB-B at a 200 mg/kg dose. The ulcer index was only 1.2%, close to the protective effect of ranitidine, which showed a 1.7% ulcer index.

### Effect on oxidative stress parameters

**Fig. 7:**
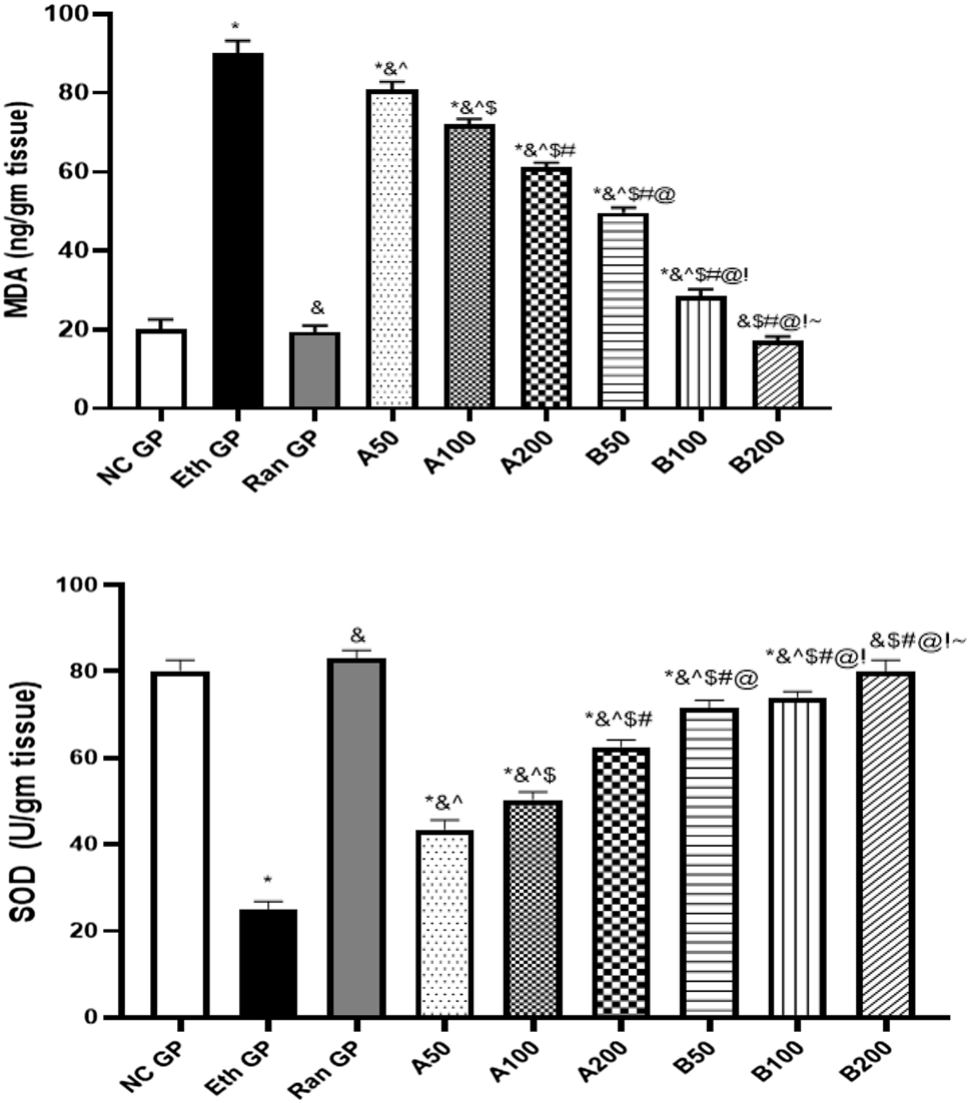
The change in the stomach tissue MDA and SOD levels after pretreatment with ranitidine or essential oils of *A. heterophylla* (**A**) or A. bidiwillii (**B**) at 3 different doses (50, 100, 200 mg/Kg, oral) 1 h before ethanol (1 ml of 95%, oral) in ethanol-induced peptic ulcer in rats. Each point represents the mean ± SD of 8 rats. Statistical significance: * is significantly different from NC GP, *P* < 0.05. & is significantly different from the Eth GP, *P* < 0.05. ^ is significantly different from Ran GP, *P* < 0.05. $ is significantly different from A50 treated group, *P* < 0.05. # is significantly different from A100 treated group, *P* < 0.05. @ is significantly different from A200 treated group, *P* < 0.05. ! is significantly different from B50 treated group, *P* < 0.05. ~ is significantly different from B100 treated group, *P* < 0.05, using one-way ANOVA followed by Tukey–Kramer multiple comparisons post-hoc test. *NC GP* normal control group, *Eth GP* ethanol treated group, *Ran GP* ranitidine treated group, *A50/100/200* EO of *A. heterophylla* at a dose of 50/100/200 mg/kg, *B50/100/200* EO of *A. bidiwillii* at a dose of 50/100/200 mg/kg

The MDA and SOD stomach tissue content of the different experimental groups are illustrated in Fig. 7. The MDA is the byproduct of lipid peroxidation, while the SOD is considered a protective agent against ROS. There was a significant increase in the stomach tissue level of MDA in the ethanol-treated group, which was about 4.5 folds compared to that of the normal control group. The concomitant administration of AH-A oil at different doses of 50, 100, and 200 mg/kg prior to ethanol administration caused mitigation in the MDA level by 11.11%, 22.22%, and 33.33%, respectively. However, intake of EO of AB-B at 50 and 100 mg/kg doses before ethanol intake caused obvious significant tackling in the MDA level by 44.44% and 66.66%, respectively, compared to the ethanol-treated group. There was no significant difference between the ranitidine group, EO of AB-B (200 mg/kg) treated groups, and the normal control group.

The results showed that the stomach tissue level of SOD decreased in the ethanol-treated group by 68.75% compared to normal rats. The treatment of rats with EO of AH-A at 50, 100, and 200 mg/kg doses elevated the SOD level by 1.8, 2, and 2.4 folds, respectively, compared to the ethanol-treated group. In comparison, treatment with EO of AB-B at the same doses mentioned above caused a marked increase in the SOD level by 2.8, 3, and 3.2 folds compared to the ethanol-treated group. The EO of AB-B at a dose of 200 mg/kg could return the SOD level to the normal range, not significantly different from the ranitidine-treated group.

### Western blot analysis

**Fig. 8:**
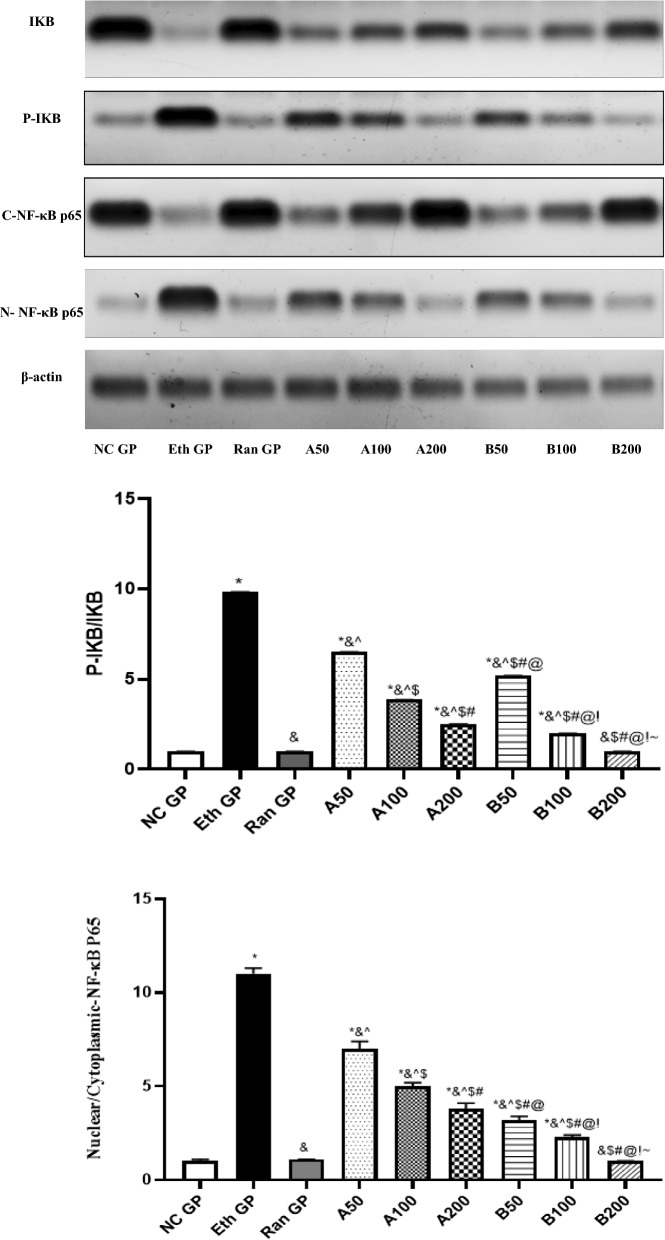
**A**, **B** The change in the stomach tissue IKB, P-IKB, N-NF-κBp65 and C-NF-κBp65 expression levels after pretreatment with Essential oils of *A. heterophylla*/*A. bidiwilli*i at 3 different doses (50, 100, 200 mg/Kg, oral) 1 h before ethanol (1 ml of 95%, oral) in ethanol-induced peptic ulcer in rats. Each point represents the mean ± SD of 8 rats. Statistical significance: * is significantly different from NC GP, *P* < 0.05. & is significantly different from the Eth GP, *P* < 0.05. ^ is significantly different from Ran GP,* P* < 0.05. $ is significantly different from A50 treated group, *P* < 0.05. # is significantly different from A100 treated group, *P* < 0.05.@ is significantly different from A200 treated group, *P* < 0.05. ! is significantly different from B50 treated group, *P* < 0.05. ~ is significantly different from B100 treated group, *P* < 0.05, using one-way ANOVA followed by Tukey–Kramer multiple comparisons post-hoc test. *NC GP* normal control group, *Eth GP* ethanol treated group, *Ran GP* ranitidine treated group, *A50/100/200* EO of *A. heterophylla* at a dose of 50/100/200 mg/kg, *B50/100/200* EO of *A. bidiwillii* at a dose of 50/100/200 mg/kg

The changes in the stomach tissue expression of IKB, P-IKB, cytoplasmic-NF-κB p65, nuclear-NF-κB p65, and β-actin in different experimental groups of our study are revealed in Fig. 8A, B. It was supposed that ethanol administration increased the level of P-IKB and decreased the IKB levels, allowing the activation and translocation of NF-κB p65 from the cytoplasm to the nucleus, as revealed by increasing the expression level of N-NF-κB p65 and decreasing that of the C-NF-κB p65.

Administration of EO of AH-A at different doses (50, 100, and 200 mg/kg) significantly decreased the P-IKB/IKB ratio with a subsequent decrease in the translocation NF-κB p65 to the nucleus, with the most obvious effect revealed in the A 200 mg/kg treated group. Concomitant treatment of the rats with EO of AB-B at the same previously mentioned doses before ethanol administration significantly mitigated the P-IKB/IKB ratio, which was accompanied by an obvious decrease in the N-NF-κB p65 as well as an increase in the C-NF-κB p65 relative expression levels, with the most obvious effect shown with the EO of AB-B 200 mg/kg treated group.

These results suggest that the extracted bark’s resin EOs of *A. heterophylla* and *A. bidwillii* could inhibit the translocation and activation of NF-κB p65 via decreasing the phosphorylation of the IKB, thus decreasing the transcription of the inflammatory cytokines, offering the given anti-inflammatory effect.

### Microscopic assessment of H & E and PAS stained sections

**Fig. 9:**
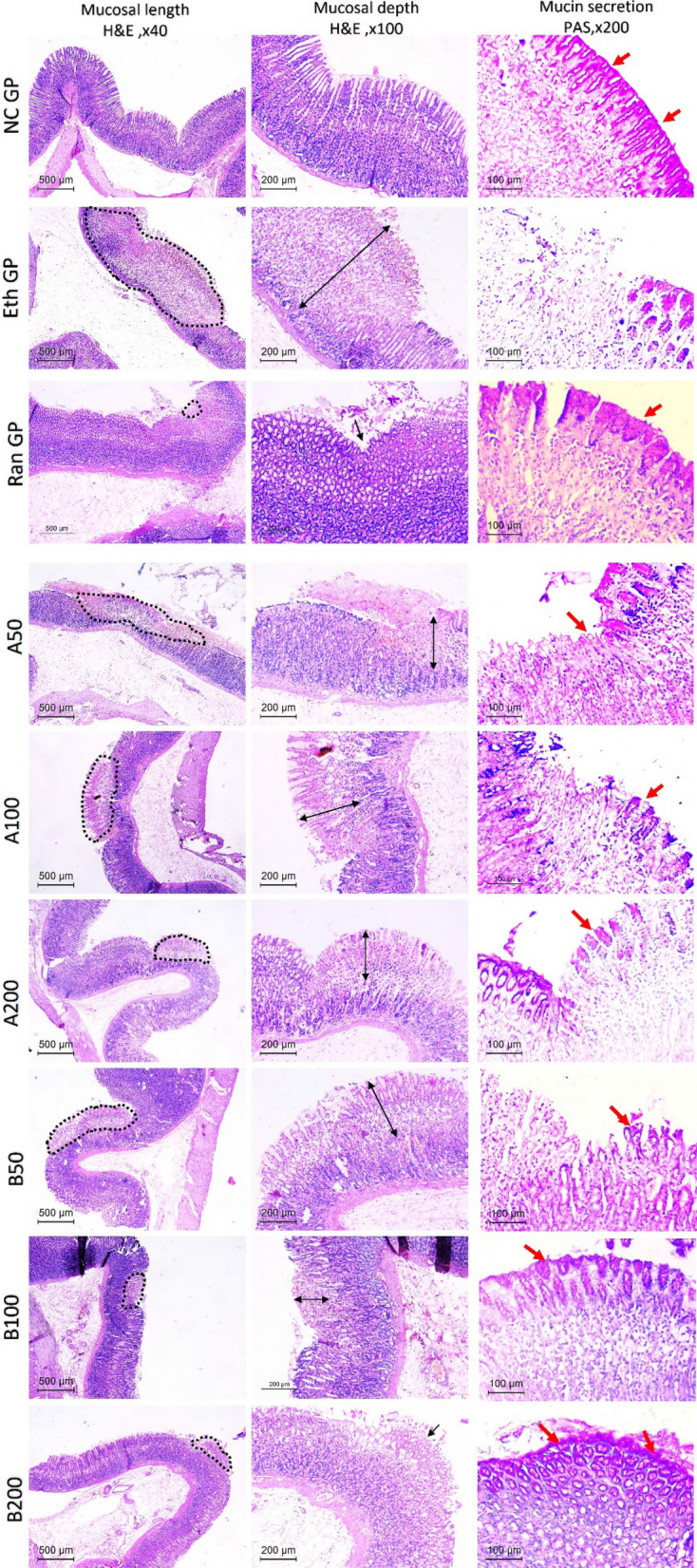
The microscopic picture of H and E stained stomachs after pretreatment with Ranitidine or Essential oils of *Araucaria heterophylla *(**A**) or bidiwillii (**B**) at 3 different doses (50, 100, 200 mg/Kg, oral) 1 h before ethanol (1 ml of 95%, oral) in ethanol induced peptic ulcer in rats. First column refers to mucosal length affected (dashed area). Normal mucosa is seen in NC GP while a large area of mucosal necrosis. Different treated groups showed decrease of necrotic areas. (H&E, × 40) Second column illustrates depth of mucosal affection in different groups (arrows). No mucosal injury in NC GP while Eth GP shows full thickness deep mucosal injury. The depth of injury decreased in treated groups to be only superfacial erosion in Ran GP and B200 (H&E, × 100). Third column shows PAS positive mucin staining in gastric pits (red arrows). Mucin is seen in the epithelial cells of NC GP while eth Gp shows mucin depletion. Restoration of mucin was seen in treated groups. (PAS, × 200). *NC GP* normal control group, *Eth GP* ethanol treated group, *Ran GP* ranitidine treated group, *A50/100/200* EO of Araucaria heterophylla at a dose of 50/100/200 mg/kg, *B50/100/200* EO of Araucaria bidiwillii at a dose of 50/100/200 mg/kg

Histopathological changes/scores in different groups in stomach-stained sections with hematoxylin and eosin (H & E)/periodic acid Schiff (PAS) are shown in Fig. 9 and Table 2. The normal control group showed intact mucosal surfaces with no ulcerations or damage. The mucosal glands showed normal architecture and lining. PAS staining showed magenta-red positive mucus vacuoles within gastric pits, especially at the neck, with faint staining in the base of the crypts.

**Table 2:** Histopathology scores of different studied groups

Groups	Mucosal length score 0 = none, 1 = < 10%, 2 = 11–20%, 3 = > 20%	Mucosal depth score 0 = no change, 0.5 = superficial, 1 = < 33%, 2 = 34–66%, 3 = > 67%	Final histopathology score length + depth (0–6)
NC GP	0	0	0
Eth GP	3	3	6
Ran GP	1	0.5	1.5
AH-A 50 GP	3	2	5
AH-A 100 GP	2	2	4
AH-A 200 GP	2	1	3
AB-B 50 GP	2	2	4
AB-B GP	1	2	3
AB-B 200 GP	1	0.5	1.5

*NC GP* normal control group, *Eth GP* ethanol treated group, *Ran GP* ranitidine treated group, *A50/100/200* EO of *A. heterophylla* at a dose of 50/100/200 mg/kg, *B50/100/200* EO of *A. bidiwillii* at a dose of 50/100/200 mg/kg

The ethanol-treated model showed wide areas of severe mucosal damage that occupied more than 20% of the gastric mucosa (score 3). The glands showed coagulative necrosis, where crypts were fragmented. Cells showed deep eosinophilic cytoplasm and karyorrhectic nuclei. These changes occupied almost the full mucosal thickness (score 3). PAS staining was lost in damaged areas indicating depleted mucus secretion.

Pretreatment by different doses of EO of AH-A showed different degrees of protection against the ameliorative effect of ethanol. At the dose of 50 mg/kg, minimal protection was seen. Wide areas of mucosal damage were still detected, with coagulative necrosis in about 50% of the mucosal thickness. PAS staining showed only focal mucin secretion in damaged areas. The protective effect was better when the dose increased to 100 mg/kg, where the damaged mucosal areas dropped to score 2 instead of score 3 in 50 mg/kg dose. However, more than two-thirds of mucosal depth were still affected.

Furthermore, the dose of 100 mg of EO of AH-A revealed moderate protection. The length of mucosal damage declined to score 2, and only one-third of mucosal depth was affected (score 1). PAS staining showed gradual restoration of mucin secretion in damaged areas.

Pretreatment by EO of AB-B showed a better protective effect against ethanol than the drug AH-A. At 50 mg/kg, the mucosal length affected by ethanol-induced damage declined to score 2, and only two-thirds of mucosal depth was affected (score 2). Mucin secretion was focally detected in damaged areas. Meanwhile, marked improvement was seen when the dose increased to 100 and 200 mg/kg. The damaged mucosal length dropped to less than 10% (score 1) and the depth of mucosa damage decreased to score 1 and 0.5, respectively. This effect was comparable to ranitidine’s protective effect, which showed only superficial erosions of the mucosal surface and focally detected mucosal damage. Mucin secretion was easily detected by PAS stain in those groups.

### Ki-67 proliferative index

**Fig. 10:**
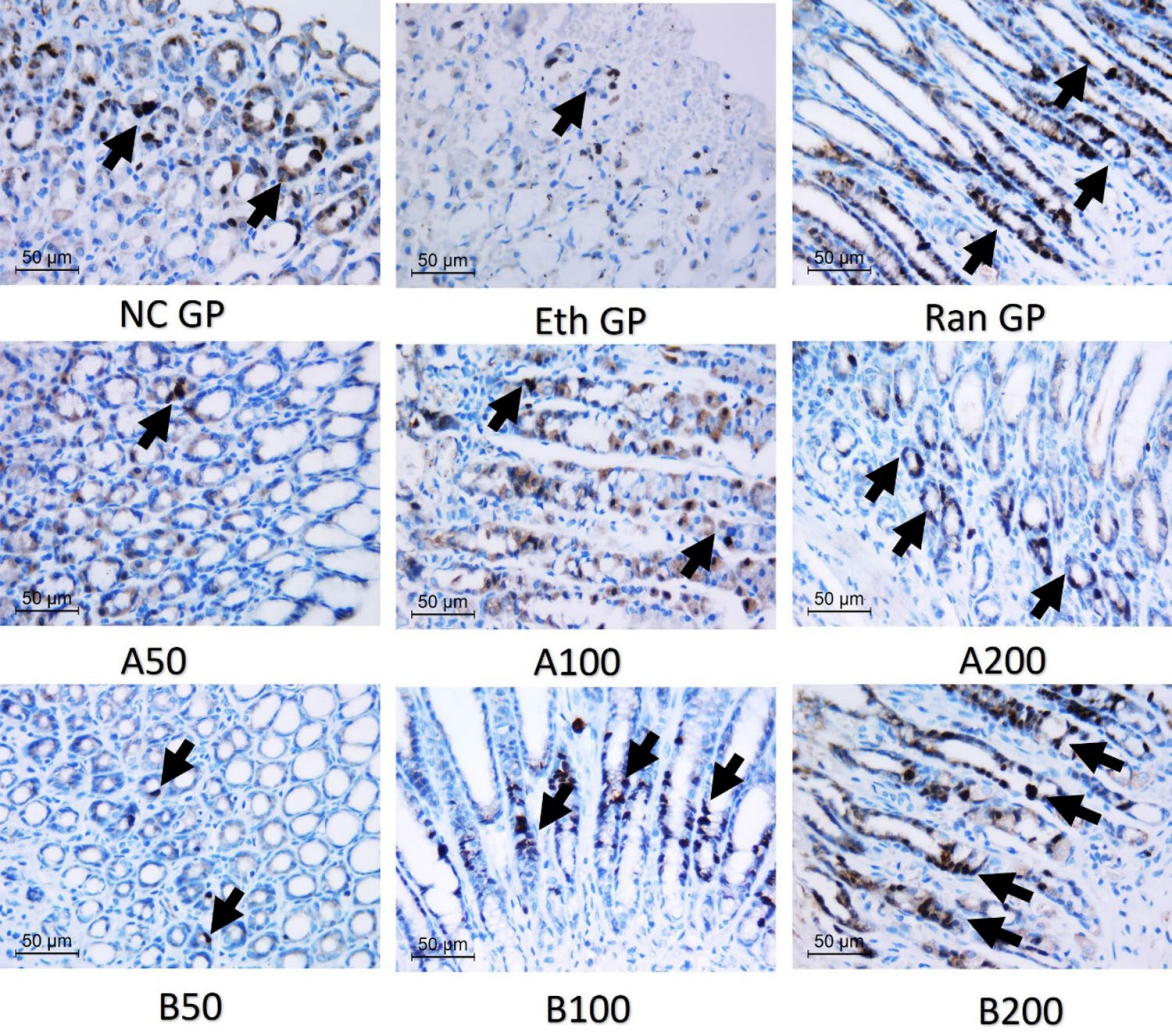
The immunohistochemical staining of Ki67 in studied groups. NC GP showing moderate nuclear positivity in neck of crypts. Eth GP showing degenerated mucosal crypts with only focal ki67 staining. After pretreatment with Ranitidine or Essential oils of *Araucaria heterophylla* (**A**) or bidiwillii (**B**) at 3 different doses (50, 100, 200 mg/Kg, oral) 1 h before ethanol (1 ml of 95%, oral), variable degrees of increased nuclear ki67 staining were noted indicating increased proliferation. Arrows refer to ki 67 nuclear stain. (ki 67 IHC, × 400). *NC GP* normal control group, *Eth GP* ethanol treated group, *Ran GP* ranitidine treated group, *A50/100/200* EO of Araucaria heterophylla at a dose of 50/100/200 mg/kg, *B50/100/200* EO of Araucaria bidiwillii at a dose of 50/100/200 mg/kg

The Ki-67 is a marker of cell proliferation, where its immunohistochemical staining in the stomachs of various studied groups is illustrated in Fig. 10. The Ki-67 proliferative index (PI) is shown in Table 3. In normal control, moderate Ki-67 nuclear positivity was seen in the neck of gastric glands. In ethanol-treated rats, Ki-67 nuclear stain was hardly seen, and PI was less than 1% due to the nearly total destruction of mucosal glands. Treatment with different doses (50, 100, and 200 mg/kg) of EO of AH-A increased the proliferative activity of mucosal crypts at the margins of damaged mucosa, indicating the increased proliferation and healing of mucosa, and the PI reached 14, 22, and 30%, respectively. At the same time, the treatment with AB-B at the same doses mentioned above increased the PI, reaching 20, 33, and 43%, respectively.

**Table 3:** Ki67 Proliferative index of different studied groups

Groups	Ki 67 proliferative index
NC GP	45%
Eth GP	6%
Ran GP	40%
AH-A 50 GP	14%
AH-A 100 GP	22%
AH-A 200 GP	30%
AB-B 50 GP	20%
AB-B 100 GP	33%
AB-B 200 GP	43%

*NC GP* normal control group, *Eth GP* ethanol treated group, *Ran GP* ranitidine treated group, A50/100/200 EO of *A. heterophylla* at a dose of 50/100/200 mg/kg, *B50/100/200* EO of *A. bidiwillii* at a dose of 50/100/200 mg/kg

### Anti-Helicobacter pylori activity

The inhibitory percentages of *H. pylori* activity at various concentrations ranging from (0.24–125 μg/mL) of AB-B, AH-A EOs and clarithromycin (standard drug) are shown in, Table 4.

**Table 4:** Anti-Helicobacter pylori effect of essential oils of *A. heterophylla* and *A. bidwillii* resin

Sample concentration (μg/mL)	Inhibition percentage		
	*A. heterophylla* (AH-A)	*A. bidwillii* (AB-B)	Clarithromycin
125	100 ± 0	100 ± 0	100
62.5	100 ± 0	100 ± 0	100
31.25	100 ± 0	100 ± 0	100
15.63	83.45 ± 2.1	100 ± 0	100
7.81	62.14 ± 1.5	100 ± 0	100
3.9	47.92 ± 0.72	78.96 ± 2.1	100
1.95	28.25 ± 0.63	59.32 ± 0.72	100
0.98	11.25 ± 1.3	43.14 ± 0.92	92.45
0.48	8.1 ± 1.4	29.82 ± 1.3	87.65
0.24	–	13.96 ± 0.58	81.35
0	0	0	0
MIC 100	31.25	7.81	1.95

Clarithromycin inhibited 100% of the bacterial growth (MIC100) when tested at 1.95 μg/mL. The EOs of both resins (AH-A and AB-B) possessed (MIC100) equal to 31.25 and 7.81 μg/mL, respectively. These results illustrated the anti-*helicobacter pylori* potential of both EOs used in this study. Moreover, they revealed that the EO of AB-B showed superior inhibitory potential on *H. pylori* growth than AH-A relative to that of the standard drug clarithromycin.

## Discussion

Despite many advances in peptic ulcer conventional pharmacological treatments, their adverse effects, interactions, and recurrence after discontinuation pursue researchers to search for suitable gastroprotective herbal medicinal plants (Sistani Karampour et al. 2019).

*Araucaria* plants possess mainly EOs that exhibit various pharmacological effects, including anti-inflammatory, antimicrobial, and anti-cancer effects (Abdelall et al. 2016; Elkady and Ayoub 2018).

In the current study, we focused on the chemical investigation of the composition of bark’s resin EO of *A. bidwillii* and *A. heterophylla* cultivated in Egypt*.* Furthermore, we assessed the gastroprotective, anti-inflammatory, and antioxidant potentials of the EOs of *A. heterophylla* and *A. bidwillii* in the ethanol-induced gastric ulcer model and their anti-*Helicobacter pylori* potential in vitro.

Chemical investigation of the studied EOs showed that they were rich in monoterpene hydrocarbons. The AH-A oil was rich mainly in α-pinene (57.79%), followed by caryophyllene (5.4%), d-limonene (4.82%), and trans-3-caren-2-ol (4.65%). While, *A. bidwillii* resin oil was enriched mainly with α-pinene (63.4%), nonane (5.21%), and trans-3-caren-2-ol (4.37%). Previous results showed that α-pinene exhibited a gastroprotective effect in experimental gastric ulcers induced by ethanol and indomethacin in mice. Furthermore, it was reported that the terpenoid components possessed antiulcerogenic and cytoprotective effects by stimulating mucus secretion via increasing prostaglandin production, improving gastric blood flow, and gastric bicarbonate **(**Baker 2020). Additionally, Monoterpenic components were reported to restore the balance between oxidant and antioxidant molecules in the gastric mucosa (Singh et al. 2010; Wannes et al. 2010).

The ethanol-induced peptic ulcer in rats is a commonly used model to assess the gastroprotective effect of new agents (Arab et al. 2015). Ethanol induces the disease via dehydration and disruption of gastric mucosal membranes, cytotoxicity, recruitment of leucocytes that enhance inflammation, and oxidative stress, as well as the reduction in the gastric blood flow and the secretion of bicarbonate and mucus (El-Maraghy et al. 2015; Park et al. 2008; Sangiovanni et al. 2013).

Many previous studies illustrated that the NF-κB activation led to an elevation in the expression of many inflammatory mediators that were all implicated in the pathogenesis of peptic ulcer (Ajayi and Olaleye 2020; Raish et al. 2021; Sidahmed et al. 2013; Su et al. 2017; Zhao et al. 2017).

Our results were in line with the previously reported studies, showing that the expression of P-IKB and N-NF-κB P65 were significantly elevated. In contrast, the expression of IKB and C-NF-κB P65 was significantly decreased in the stomach of untreated peptic ulcer rats. We could suggest that the phosphorylation of IKB enhanced the translocation of NF-κB P65 from the cytoplasm to the nucleus.

Additionally, there was a significant increase in the serum levels of TNF-α and IL-1β in untreated peptic ulcer rats. This can be conveyed by the increase in their transcription via the observed activation in the NF-κB.

Pretreatment of rats with EOs of AH-A and AB-B at different doses (50, 100, and 200 mg/kg) one hour before ethanol administration significantly inhibited the phosphorylation of IKB and, subsequently, the nuclear translocation of NF-κB, as well as decreased the serum levels of TNF-α and IL-1β.

The most significant inhibition in the expression of P-IKB and N-NF-κB p65 was seen in the AB-B EO-treated group, which showed a more prominent effect than AH-A EO. The most obvious effect was seen in the AB-B EO-treated group at a 200 mg/kg dose that was not significantly different from ranitidine (used as a standard drug). It was previously reported that inhibiting the NF-κB and its downstream targets had a role in the healing of peptic ulcers (Zhao et al. 2017). Furthermore, it had been illustrated before that the crude EO of the Egyptian *A. heterophylla* at a dose of 200 mg/kg reduced the levels of proinflammatory cytokines (TNF-α, IL-6, and IL-1β), paw edema, and rectal temperature in a carrageenan-induced rat model (Elshamy et al. 2020a, b). At the same time, the shoots’ EO of Egyptian *A. bidwillii* possessed the same effect as *A. heterophylla* in the same animal model with the additional capability of attenuating the immunohistochemical NF-κβ levels in paw tissues (Abdelhameed et al. 2021).

Oxidative stress plays an important role in the pathophysiology of peptic ulcers. Ethanol could increase the state of imbalance between oxidant and antioxidant molecules, shifting the balance to favor the oxidative stress state (Zhou et al. 2020). Our results favor the study mentioned above, showing obvious elevation in the MDA stomach tissue level and a decrease in the antioxidant enzyme SOD level in the untreated peptic ulcer rats.

Ranitidine pretreatment significantly prevented the induction of an oxidative stress state. This was obvious by a significant reduction in the stomach MDA level and elevation in the SOD level. Pretreatment of rats with EO of AH-A or AB-B at different doses (50, 100, and 200 mg/kg orally) significantly reduced the tissue MDA levels and elevated SOD. It was proposed previously that the extracts of Egyptian *A. bidwillii* and *A. heterophylla* could mitigate the progression of inflammatory conditions related to oxidative stress, such as in COVID-19 (El-Hawary, Rabeh et al. 2021).

The effect of EO of AB-B was the most prominent at a dose of 200 mg/kg and was similar to the effect seen in the ranitidine-treated group. This highlighted the antioxidant capabilities of the EOs of both species, with the superiority of the EO of AB-B over that of AH-A.

Previously, it was reported that the elevation in the ROS seen in the peptic ulcer untreated rats can subsequently lead to activation of the NF-κB signaling pathway, aggravating the inflammatory condition **(**Yoo et al. 2020**)**. We could suggest that the antioxidant capabilities of both EOs could inhibit the activation of the NF-κB pathway, mitigating the production of inflammatory cytokines. This highlighted the gastroprotective role of these EOs via offering antioxidant and anti-inflammatory potentials.

The macroscopic assessment of the collected stomachs revealed a significant increment in the percentage of ulcer area in untreated peptic ulcer rats, reaching 26% of the total area. The same results were confirmed by previous studies (Li et al. 2021; Rahman et al. 2020).

Our results illustrated that treating rats with EOs of AH-A or AB-B in the previously mentioned doses before alcohol administration reduced the percentage of ulcer area and elevated the ulcer protection percentage. The most obvious protective effect was seen in rats treated with AB-B oil at a dose of 200 mg/kg, which was not significantly different from the effect of ranitidine as a standard drug. It was reported that the stem’s resin extract of *A. heterophylla* offered a significant reduction in the percentage of ulcer area and elevation in the ulcer protection percentage in the ethanol-induced peptic ulcer model (Abdel-Sattar et al. 2009).

The histopathological changes observed in H & E-stained stomach sections in the ethanol-treated model showed wide areas of severe mucosal damage, vascular congestion, submucosal edema formation, and inflammation. Our histopathological findings in untreated peptic ulcer rats were similar to the findings observed previously (Guzmán-Gómez et al. 2018; Rahman et al. 2020). The PAS staining was totally lost in damaged areas indicating depleted mucin secretion in the stomachs of untreated peptic ulcer rats (Júnior et al. 2020). Even though pretreatment with EOs of AH-A or AB-B at different doses hindered the aforementioned histopathological changes observed in H/E and PAS-stained stomach sections, offering a gastroprotective effect. The most showed gastroprotective effect was offered by AB-B oil (200 mg/kg) which was not significantly different from ranitidine. A previous report showed that shoots’ EO of Egyptian *A. bidwillii* decreased the histopathological changes and inflammation seen in hind paws in a carrageenan-induced rat model (Abdelhameed et al. 2021).

The Ki-67 is a nuclear protein related to cell proliferation, which is an important issue for healing (Ajayi and Olaleye 2020). In untreated peptic ulcer rats, Ki-67 nuclear stain was hardly seen, and the proliferative index was less than 1% due to the nearly total destruction of mucosal glands, which was in line with a previous study showing the same effect (Brito et al. 2018). Treatment at different doses of EOs of AH-A or AB-B increased the proliferative activity of mucosal crypts at the margins of damaged mucosa, indicating increased mucosa healing. This suggested that the gastroprotective potential of the obtained EOs could be mediated by enhancing cell proliferation that aids in the healing process. The most obvious effect was seen in the rats treated with EO AB-B at a dose of (200 mg/kg).

*Helicobacter pylori*, a gram-negative bacterium, can affect the stomach leading to oxidative stress state which plays main role in the pathogenesis of peptic ulcers (Wang et al. 2022). The inhibitory potential of both EOs against *H. pylori* activity was assessed in vitro. Our results illustrated that the effective inhibitory pattern on *H. pylori* growth was more obvious in the EO of AB-B than that of AH-A oil as compared to clarithromycin as a standard drug. This could be explained due to the presence of monoterpenoids in their chemical composition that had been previously reported to possess bactericidal effect against *H. pylori* (Korona-Glowniak et al. 2020; Périco et al. 2020). Also, previous study demonstrated the anti-helicobacter potential of α-pinene in the EO of *Pistacia atlantica* (Memariani et al. 2017).

Many studies suggested that anti-microbial effect of terpenoids was due to their ability to affect the microbial membrane integrity and subsequently impair the intracellular PH (Prabuseenivasan et al. 2006; Sikkema et al. 1994).

## Conclusion

In conclusion, this research is considered the first study on the phytoconstituents of the bark’s resin EO of *A. bidwillii* cultivated in Egypt. Additionally, it is the first study that compared chemical profiling of the resin oil composition of *A. heterophylla* and *A. bidwillii.* Both EOs had promising gastroprotective effects revealed by their anti-*Helicobacter pylori* potential in vitro as well as antioxidant and anti-inflammatory activities in vivo. We could propose that the gastroprotective effect offered could be conveyed by the modulation of ROS/NF-κB/inflammatory cytokines as well as increasing the expression of Ki-67, leading to an increase in the cell proliferation and healing processes. It was observed that the gastroprotective effect offered by EO of *A. bidwillii* was more prominent than that of *A. heterophylla*. These results suggest using EO AH-A or AB-B after stopping conventional peptic ulcer treatments; to protect these patients from recurrence. Further studies are required to test their ability to treat gastric ulcers alone or in combination with conventional treatments, with verifying the type of interaction if used in combination.

## Data Availability

All data generated or analyzed during this study are included in this article.
